# Cumulative impact of anti-sea lice treatment (azamethiphos) on health status of Rainbow trout (*Oncorhynchus mykiss*, Walbaum 1792) in aquaculture

**DOI:** 10.1038/s41598-019-52636-1

**Published:** 2019-11-07

**Authors:** Josip Barisic, Stuart Cannon, Brian Quinn

**Affiliations:** 1 Aquaculture Health Laboratory, School of Health and Life Sciences, University of the West of Scotland, Paisley, PA1 2BE Scotland, UK; 2 Ruđer Bošković Institute, Division of Materials Chemistry, Laboratory for biotechnology in aquaculture, Zagreb, Croatia; 3 Kames Fish Farming Ltd., Kilmelford, PA34 4XA Scotland, UK

**Keywords:** Nephrons, Ichthyology

## Abstract

Despite its widespread use in aquaculture, the impact of chemical anti-sea lice treatment on salmonids following application in a commercial farm has not been previously reported. This work reports the cumulative effect of three consecutive anti-sea lice treatments using azamethiphos on the health status of aquaculture reared rainbow trout through the investigation of clinical chemistry, histopathology and proteome expression. The serum biomarkers showed decreasing trends in total protein, albumin and potassium concentrations and an average increase of total bilirubin and phosphate concentration towards the end of the treatment period. Principal component analysis clearly distinguished correlated pairs of biomarkers and also demonstrates a shift from acute to chronic effects as treatment progresses. Proteomic analysis confirmed alterations of proteins involved in clot formation, immune reaction and free heme binding. Tissue damage after the series of delousing treatments, exhibited increased deposits of hemosiderin. Results from this study suggest an impact of azamethiphos on trout health through intravascular haemolysis and consequently from pathophysiologic process of haemoglobin metabolism and its products, causing chronic kidney injury from iron deposits. This is the first report to demonstrate in fish the impact of active iron accumulation in different organs from physiological processes that can seriously impair normal function.

## Introduction

Large marine trout aquaculture is a rather new but significant food industry in Europe with a production of 153,954 tonnes in 2016^1^, roughly a tenth the size of European salmon production (1,488,434 tonnes). This is why today, the various aquaculture industries are recognized as major forms of primary production having important economic, social and environmental impact. Like in all other farm animal husbandry, disease represents an important barrier to production that must be addressed. However, in the marine environment, sea lice infestations with caligid copepods *Lepeophtheirus salmonis* and *Caligus elongatus* are commonly found in open sea cage systems and represent the primary cause of economic loss and impaired animal welfare^2,3^. The total costs including anti-sea lice treatments costs and losses resulting from the decline in fish growth, were estimated to be USD $480 M annually for the global salmonid aquaculture industry in 2006^4^. More recently, the impact on Norwegian aquaculture alone for 2011 is estimated at USD $436 M, excluding the costs from Chile, UK, Canada, Ireland, Faeroes and USA salmonid aquaculture^5^. Therefore, vigorous attempts are underway to reduce sea lice numbers by various treatment methods based on chemical medication, mechanical removal or environmental manipulation.

Chemical measures to control sea lice populations include the application of chemical compounds such as organophosphates (azamethiphos in particular), synthetic pyrethroids (deltamethrin and cypermethrin), enamectin benzoate and hydrogen peroxide in the form of a bath treatments or medicated feed^6^. Azamethiphos (AZA) is an organophosphate insecticide and the active ingredient in the powder formulation Salmosan^®^ used for bath treatments against sea lice, with a recommended dosage of 0.1 to 0.2 mg L^–1^ for up 1 h exposure. Azamethiphos is registered for use in salmon aquaculture in Norway, Ireland, Scotland and Chile. Although commonly used in salmon aquaculture it was also shown to be affective against sea lice in trout^7,8^. Organophosphorus compounds are not effective against juvenile stages, with only mature sea lice (stages pre-adult to adult) affected during treatment. Therefore, in order to ensure complete eradication of the lice, treatments may need to be repeated after 10 to 20 days and again after a further 14 days. Azamethiphos is a neurotoxic agent, causing acetylcholinesterase (AChE) inhibition^9^. The function of AChE is to mediate the hydrolysis of the nurotransmitter acetylcholine, which subsequently terminates transmission through the synaptic membrane. AChE is also present in membranes of red blood cells, but its function is unknown^10^. The reported acute 96-h LD_50_ for rainbow trout exposed to azamethiphos is 0.2 mgL^−1^^11^. Atlantic salmon tolerates a single 1-hour exposure of 0.5 mgL^−1^ and three 1-hour doses of 0.3 mgL^−1^ repeated at weekly intervals. However, some mortalities were detected in fish exposed to 1.0 mgL^−1^^12^. Therefore, we hypothesized that repeated minor exposures may cause a cumulative poisoning effect due to acetylcholinesterase inhibition, which may be reflected in the fish health status. Acetylcholinesterase levels have been found to return to normal several weeks following exposure^12^.

Fish welfare has become increasingly important in aquaculture and continuous health monitoring during sea lice treatments is essential to ensure fish wellbeing. Clinical chemistry analysis has been previously undertaken in rainbow trout^13^ and has proven a useful tool to analyze the health status of fish following pollution exposure^14^, feed trials^15,16^, disease monitoring and diagnosis^17,18^, toxicological studies^19,20^ and to investigate pathophysiology^21^. There is therefore a need to investigate the clinical impact of Azamethiphos under commercial aquaculture conditions to detect the specific organ injury, potentially leading to organ impairment.

Depending on their usage, different sets of biomarkers can be used to monitor an impact on a specific target organ. The only biomarker previously used to measure the impact of sea lice treatment is the AChE assay, which is specific for organophosphate toxicity and is measured in brain tissue homogenates^22^. We hypothesize that consecutive AZA treatments cause a cumulative effect on fish health through a cascade of red blood cell intravascular degradation and accumulation of oxidized ferric (Fe^3+^) in different tissues of the rainbow trout. This can lead to kidney damage, as nephrotoxicity is evident in animal and human cell models following exposure to organophosphates^23^. Therefore, the objectives of the present study were to investigate the possible causes of impaired fish health after consecutive azamethiphos sea lice treatments under commercial aquaculture conditions using a variety of clinical chemistry assays. Moreover, the goal was to link specific organ injury response with in-depth tissue structure analysis. Finally, by using rapid methods of blood biochemistry analyses combined with histopathology and proteome expression, our aim was to investigate the possible pathophysiological mechanisms behind the impacts observed after repeated anti sea lice treatments.

## Materials and Methods

### Study location, sea lice treatments, sampling regime and water quality

Samples were provided by a commercial trout farm (Kames Fish Farming Ltd.) based in Loch Melford, western Scotland. A population of all-female diploid (AquaGen) Rainbow trout from a 70 m pen were routinely monitored for the presence of sea lice *Lepeophtheirus salmonis* and *Caligus elongatus*. The cage contained approximately 22 000 fish, with a mean body weight of 2.5 kg at a stocking density of 11.7 kg/m^3^. In summer 2017, after a sea lice infestation (>3 lice/fish), the pen under investigation was treated using Salmosan^®^Vet (azamethiphos, 500 mg/g powder) bath for three consecutive treatments, with 12 days between first and second treatment and 21 days between the second and third treatment. An enclosed plastic skirt (1800 m^3^) was used, and fish were treated with 0.2 ppm of azamethiphos for 45 min with additional oxygen injection as suggested by the manufacturer. Sampled fish were randomly selected by hand-netting and anesthetized with 100 mg/L MS-222 (Sigma) following manufacturer’s instructions. A total of 30 fish were sampled for sea lice counts and blood collected for husbandry health analysis immediately before the bath treatment (representing reference values) and 24 hours, 48 hours, 4 days and 10 days post-treatment. Pre-treatment fish were sampled from the pool of crowded fish, in order to eliminate the effect of stress. Blood samples were taken from the caudal vein by using lateral approach. After withdrawal, bloods were placed in an empty Eppendorf tube and were allowed to clot for 3 hours at +4 °C. Following this time, the bloods were centrifuged for 10 minutes at 1300 × *g*, the serum was separated and stored at −80 °C until analysis within three months. Fish (n = 5) were selected for routine histopathological analysis of gills, liver, spleen and kidney tissue before the first treatment and 10 days after the third azamethiphos treatment. Daily values of hydrographic parameters were measured at the site using a HI 9828 Multiparameter probe (Hanna Instruments Srl, Italy) for salinity (psu), dissolved oxygen (DO, %), seawater temperature (T, °C), and pH.

### Serum clinical chemistry analysis

All serum samples were analysed on the Daytona RX (Randox Laboratories Ltd., Crumlin, UK) clinical chemistry analyser at 37°C, using commercial kits (Randox Laboratories Ltd., UK) following the producer’s instructions. Biochemical analysis was undertaken for 13 biomarkers (alkaline phosphatase (ALP), alanine aminotransferase (ALT), total protein (TP), albumin (ALB), total bilirubin (TBIL), lactate dehydrogenase (LDH), creatinine (CREA), inorganic phosphorus (P), potassium (K), iron (Fe), copper (Cu), haemoglobin (HB) and glucose-6-phosphate dehydrogenase (G-6-PD). Haptoglobin (HP) concentrations were quantified using the Fish Haptoglobin ELISA Kit (My BioSource, USA).

### Histopathological analysis

Routine histological analyses was undertaken. In brief, tissues were preserved in 4% buffered formalin for 24–72 h, transferred to 50% ethanol before being dehydrated through 70%, 96% and absolute ethanol, cleared in xylene and soaked in paraffin wax using an automatic Excelsior AS processor (Thermo Fisher Scientific Inc., USA). Paraffin block sections were cut at 2 μm using a microtome Microm HM 355 S (Thermo Fisher Scientific Inc., USA), dewaxed overnight in at 60 °C and stained with modified Harris haematoxylin and Young’s eosin stains (Thermo Fisher. Scientific Inc., USA). To demonstrate ferric iron in tissue sections, Pearls Prussian blue histochemical method was used (Merck, Germany). After staining, sections were dehydrated in increasing concentrations of ethanol (70–100%), cleared in xylene and mounted in Biomount DPX (Biognost, Croatia). Microphotographs were taken using Axio Scan.Z1 scanning light microscope (Zeiss, Germany) and were edited using ZEN 2.3 imaging software. Severity of histopathological alterations was assessed using semi-quantitative approach as control (0), mild (+), moderate (++) and severe (+++).

### Serum proteome analysis

Proteomic analysis was undertaken on six serum samples consisting of two pooled response replicates from 5 fish, sampled before, 48 h and 4 days after the second azamethiphos treatment. The serum was depleted of highly abundant albumin using salt-ethanol precipitation, alkylated and digested by trypsin following the procedure described in Bilić *et al*.^24^. Next, one-hundred micrograms of peptide from each sample was labelled with Amine-Reactive Tandem Mass Tag Reagents (Thermo Fisher. Scientific Inc., USA) according to the protocol supplied by the manufacturer. The labelled peptides were solubilized in 2% acetonitrile with 0.1% trifluroacetic acid, combined and fractionated on a nanoflow uHPLC system (Thermo RSLCnano) with online analysis by electrospray ionisation (ESI) mass spectrometry on an Orbitrap Elite mass spectrometer (Thermo Fisher Scientific Inc., USA). Samples were combined at equal amounts into 2 sets, the first consisting of 1 internal standard, 2 pooled samples before and 2 pooled samples 48 h after the treatment and second consisting of 1 internal standard and 2 pooled samples 4 days after the treatment. Acquired MS/MS spectra were analyzed for protein identification and quantification using Proteome Discoverer software 2.1 (Thermo Fisher Scientific Inc., USA). Protein identifications were assigned using the Mascot algorithm against the teleost taxonomical group in the NCBI database, allowing a mass tolerance of 10 ppm for the precursor and 0.6 Da for MS/MS matching. Quantification was performed using abundances of reporter ions based on signal to noise ratio values or intensity. Abundances ratios were obtained for each protein by comparing with values of the corresponding internal standard and then used to calculate average fold change ratio groups. To investigate the relationship among different time-points and different proteins with respect to abundance, the dataset was analysed using hierarchical cluster analysis based on Euclidean distance as previously described in Braceland *et al*.^25^ and the results were illustrated in the form of a heat map using ArrayStar software (DNASTAR, USA).

### Data processing and statistical analysis

All statistical analyses was performed using multiple linear regression operating on R language package (version i386 3.0.3, R Foundation for Statistical Computing, Austria) and divided into univariate and multivariate parts. The univariate part included analysis of variance with *post – hoc* tests on each variable, while multivariate analysis was performed by principal component analysis (PCA) on selected variables. A Box–Cox method was conducted to find the best transformation of the variables in terms of the normal distribution. In addition, this kind of transformation homogenizes the variances of different groups of data, linearizes the associations between skewed variables and reduces the problem of extreme outliers found within the data. The comparison of biochemical parameters in trout serum between different time points before and after the treatment was performed using a Welch’s ANOVA and *post-hoc* multiple comparisons using nonparametric Games – Howell test with level of significance *p* < 0.05. Groups that were not significantly different were assigned the same letter of the alphabet, as reported in tables and diagrams. To test the differences between overall averages of three treatments for each endpoint, more conservative *p* < 0.001 level of significance was used. Outliers were detected in multivariate fashion using Mahalanobis distance with robust covariance matrix estimation (MCD – minimum covariance determinant) and a 0.05 cut-off value, with outlying cases removed listwise. Principal component analysis (PCA) was performed on clinical chemistry endpoints with the first two principal components, accounting for 42.7% of total variance (PCA1–25.3%, PCA2–17.4) retained for interpretation.

### Ethical statement for animal experimentation

This study was approved by University of the West of Scotland ethics committee. Samples were collected by the fish farm as part of routine fish husbandry practice and therefore not regulated by the Home Office. Fish were anaesthetised before a blood samples was taken and euthanized before tissue samples were taken using MS-222 (Sigma) following the manufacturer’s instructions. All methods were performed in accordance with the relevant guidelines and regulations covered by EC directive 2010/63/EU “Protection of animals used for scientific purposes”.

## Results

### Seawater conditions and fish general health

The seawater temperature ranged between 14.1 °C and 15.0 °C during the study period with an average salinity was 33 ppt at the surface and 34 ppt at 5 m depth. The pH values were between 7.8 and 8.2 and the dissolved oxygen concentration was in range of 8.1 to 9.0 mg/L. The trout used in the study had an average fork length of 54.0 ± 4.5 cm and body weight of 2.5 ± 0.42 kg. Adult female lice numbers were >3 per farmed fish prior to treatment. Before the first treatment, trout were assessed by the farmers to be in good nutritional condition and vigorous. At the end of the third exposure, trout displayed obvious weakened condition with lower appetite and behavioural changes observed by the farmers. There were no immediate mortalities after the first two azamethiphos exposures, whereas mortalities occurred at the rate of 1% after the third exposure.

### Serum clinical chemistry

**Figure 1:**
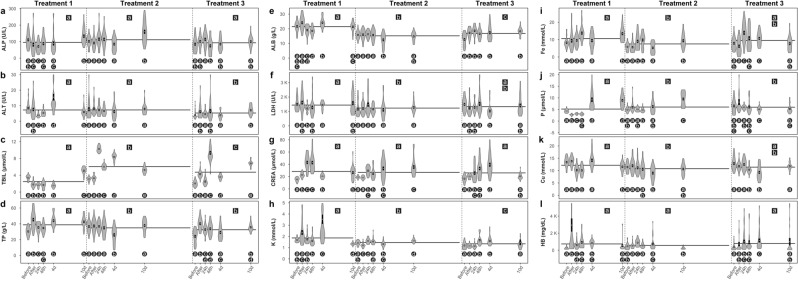
Violin graphs showing concentrations and activity of different clinical chemistry endpoints with mean and standard error (SE), before and after three consecutive azamethiphos treatments. Different letters (in circles) indicate significant differences (*p* < 0.05) for each clinical chemistry endpoint, between different time points within each treatment. Different letters (in squares) presented on mean lines show level of significant difference *p* < 0.001 between treatments for each clinical chemistry endpoint. Abbreviations: ALP (alkaline phosphatase), ALT (alanine transaminase), TBIL (total bilirubin), TP (total protein), ALB (albumin), LDH (lactate dehydrogenase), CREAT (creatinine), K (potassium), Fe (iron), P (phosphate), Cu (Copper), HB (hemoglobin).

The complex relationship between clinical chemistry biomarker expression before, immediately after, 24 h, 48 h, 4 days and 10 days after each of the three individual 45 min exposures to azamethiphos (as Salmosan^®^) is illustrated in Fig. 1. This graph uses violin plots (mean ± SE) with different letters to indicate significant differences (*p* < 0.05) and shows all three consecutive treatments over a time line. Differences in biomarker expression following treatment in each exposure are compared with the data collected before the treatment and between the different sampling points.

In summary, in an early acute reaction, significant increases in TP, CREA and HB expression were observed immediately after the first treatment (Fig. 1d,g,l), with TBIL, TP, ALB and CREA (Fig. 1c,d,e,g) also significantly increased immediately after the third treatment. At 24 h and 48 h after the first treatment, in a late acute reaction, median activity of ALT and Cu concentration was significantly lower than before the treatment (Fig. 1b,k), while lower activity of ALP and LDH occurred only 24 h after the treatment (Fig. 1a,f). The same time points in the second treatment showed significant increases in TBIL, CREA, Fe and HB (Fig. 1c,g,i,l). After the third treatment higher concentrations of TP, ALB, CREA, Fe and HB were present at both late acute reaction time points (24 h and 48 h) (Fig. 1d,e,g,i,l).

**Figure 2:**
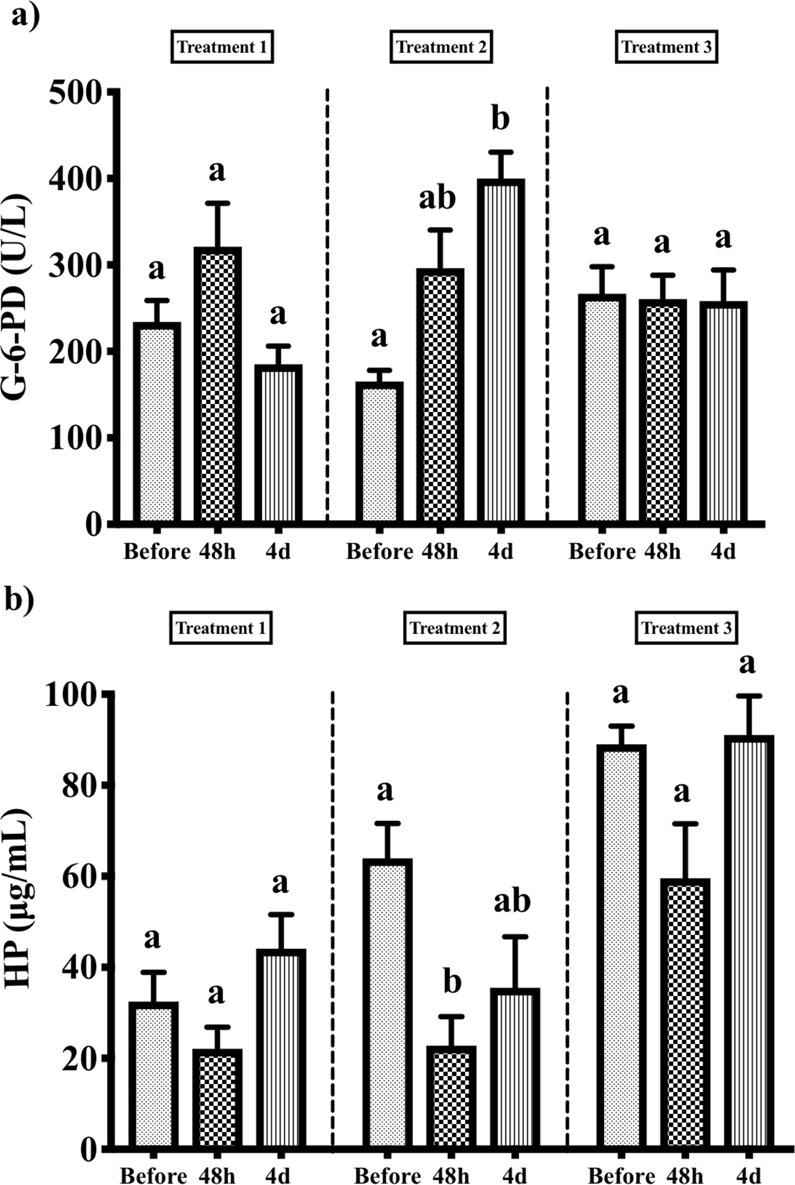
(**a**) Serum activity of glucose 6 phosphatase dehydrogenase (G-6-PD) and (**b**) haptoglobin (HP) concentration during three consecutive azamethiphos treatments. Different letters indicate significant differences between time points in each treatment (*p* < 0.05).

The time points 4 and 10 days post treatment represent a prolonged reaction. Following the first treatment, ALT activity was significantly higher after 4 days and significantly lower 10 days post treatment (Fig. 1b). Concentrations of TP, CREA and P were statistically higher when compared to pre-treatment samples (Fig. 1d,g,j), while K concentrations were higher at 4 days and lower after 10 days post treatment (Fig. 1h). Concentrations of TBIL, CREA and P were significantly higher after the second treatment (Fig. 1c,g,i). Following the third treatment, ALB levels were significantly higher in both time points (Fig. 2a), while concentrations of CREA, K and Fe were higher 4 days post treatment (Fig. 1g–i) and ALT activity and concentrations of TBIL and TP were higher 10 days post treatment (Fig. 1b–d).

In addition, the data from all time points per treatment for each biomarker were pooled to enable comparisons between treatments presented as a mean line with the level of significant difference set as *p* <  0.001, presented as letters in a box. From this pooled data it is can be seen that ALT activity significantly decreased following the third treatment and concentrations of TP, ALB and K significantly decreased after the second and third treatments (Fig. 1b,d,e,h). Concentrations of TBIL and P were significantly increased after the second and third treatments (Fig. 1c,j), while concentrations of Fe and Cu were significantly lower following the second treatment (Fig. 1i,k).

Azamethiphos exposure had a significant impact on G-6-PD activity and HP concentration (results show 3 time points per treatment) (Fig. 2). During the second treatment, a significant increase in G-6-PD enzyme activity was seen from before (165.15 ± 12.94 U/L) to 4 days post treatment (399.9 ± 30.48 U/L), while no significant change was observed during the first and third treatments (Fig. 2a). Similarly, during the second treatment HP concentration showed a significant decrease from 63.96 ± 7.65 μg/mL before the treatment to 22.78 ± 6.36 μg/mL at 48 h post treatment (Fig. 2b).

**Figure 3:**
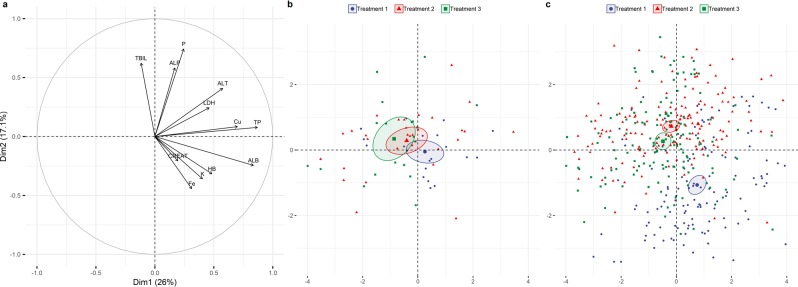
(**a**) Principal Component Analysis (PCA) bi-plot of serum endpoints (arrows). (**b**) Principal Component Analysis (PCA) bi-plot from of three control time points (before) within consecutive azamethiphos treatments. (**c**) Principal Component Analysis (PCA) bi-plot of three consecutive azamethiphos treatments. Abbreviations: TBIL (total bilirubin), P (phosphate), ALP (alkaline phosphatase), ALT (alanine transaminase), LDH (lactate dehydrogenase), Cu (copper), TP (total protein), ALB (albumin), HB (hemoglobin), K (potassium), CREAT (creatinine), Fe (iron).

**Figure 4:**
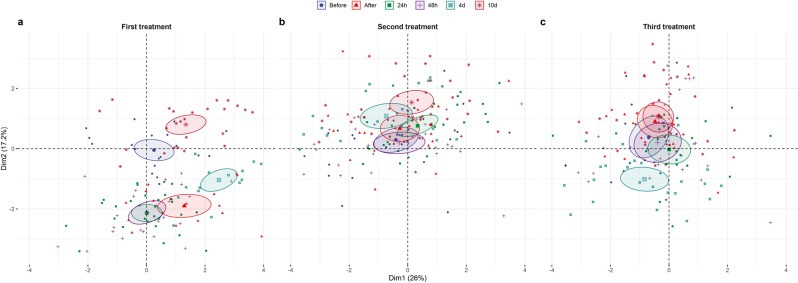
(**a**) Principal Component Analysis (PCA) bi-plot from the first azamethiphos treatment, (**b**) second azamethiphos treatment, (**c**) third azamethiphos treatment, with ellipses responding to different time points.

Principal Component Analysis (PCA) bi-plot results of serum endpoints are displayed in Figs 3, 4. Depicting clinical chemistry endpoints as arrows in the space spanned by the first two PCs, relations between variables can be discerned (Fig. 3a). Arrows that point along the same line imply high correlations between corresponding endpoints: pointing the same direction means highly positive correlation, while opposite direction stands for a negative correlation. Arrows that are approximately perpendicular imply low or no correlation between endpoints. Also, length of the arrow denotes the quality of representation for the corresponding variable in the space of first two PCs: arrows that extend close to the unit circle are very well explained by the two PCs, while short arrows imply that the variable does not have a good representation in the first two dimensions. In this analysis, the first PC is dominantly composed of variables TP, ALB and Cu concentrations, where ALT activity also has some contribution. The second PC is dominated by concentrations of P and TBIL and ALP activity, with some contribution from concentrations of Fe and K, but in the opposite direction. ALT activity also contributes to this dimension. These two PCs roughly define two groups of variables that are highly correlated within the group but have low correlations between the two groups. ALT activity is the variable that has moderate correlation with both PCs. PCA diagram Fig. 3b with data corresponding to measurements taken before administering the chemical, shows that from the first treatment to the last, there was a shift in the negative direction of PC1. PCA diagram Fig. 3c shows all clinical chemistry data grouped by the treatment criteria, with confidence ellipses drawn for each treatment defining a confidence region for the two-dimensional mean of the treatment, with all time points. Higher separation of ellipses, as shown on the graph, implies that the means of those groups of data are significantly different. This diagram with data grouped by treatments shows that there is a significant shift of location between the overall mean of the first treatment and the means of the other two. The shift is mostly along the PC2 with some change in PC1. This corresponds with an increase in variables TBIL and P concentrations and a decrease in concentrations of TP and ALB.

PCA diagram (Fig. 4a) from the first azamethiphos treatment showed well separated time point groups, with measurements after treatment, up to 48 h later, showing a significant shift in the negative direction of PC2 compared to measurements before treatment. After 4 days, there is a large increase, mostly in variables aligned with PC1, including ALT (Fig. 3a). After 10 days, location is shifted closer to measurements recorded before the treatment. PCA diagram (Fig. 4b) from the second azamethiphos treatment showed very poor separation of time point groups, where ellipses for measurements up to 48 h are mostly overlapped. Only at 4 days after the treatment there was some significant separation, mostly in the positive direction of PC2. The PCA diagram (Fig. 4c) from the third treatment showed a similar pattern to the second treatment. However, at 4 days after the treatment, there was a more pronounced location shift in the negative direction of PC2, while after 10 days this location has shifted closer to measurements recorded before the treatment.

### Histopathological analysis

**Figure 5:**
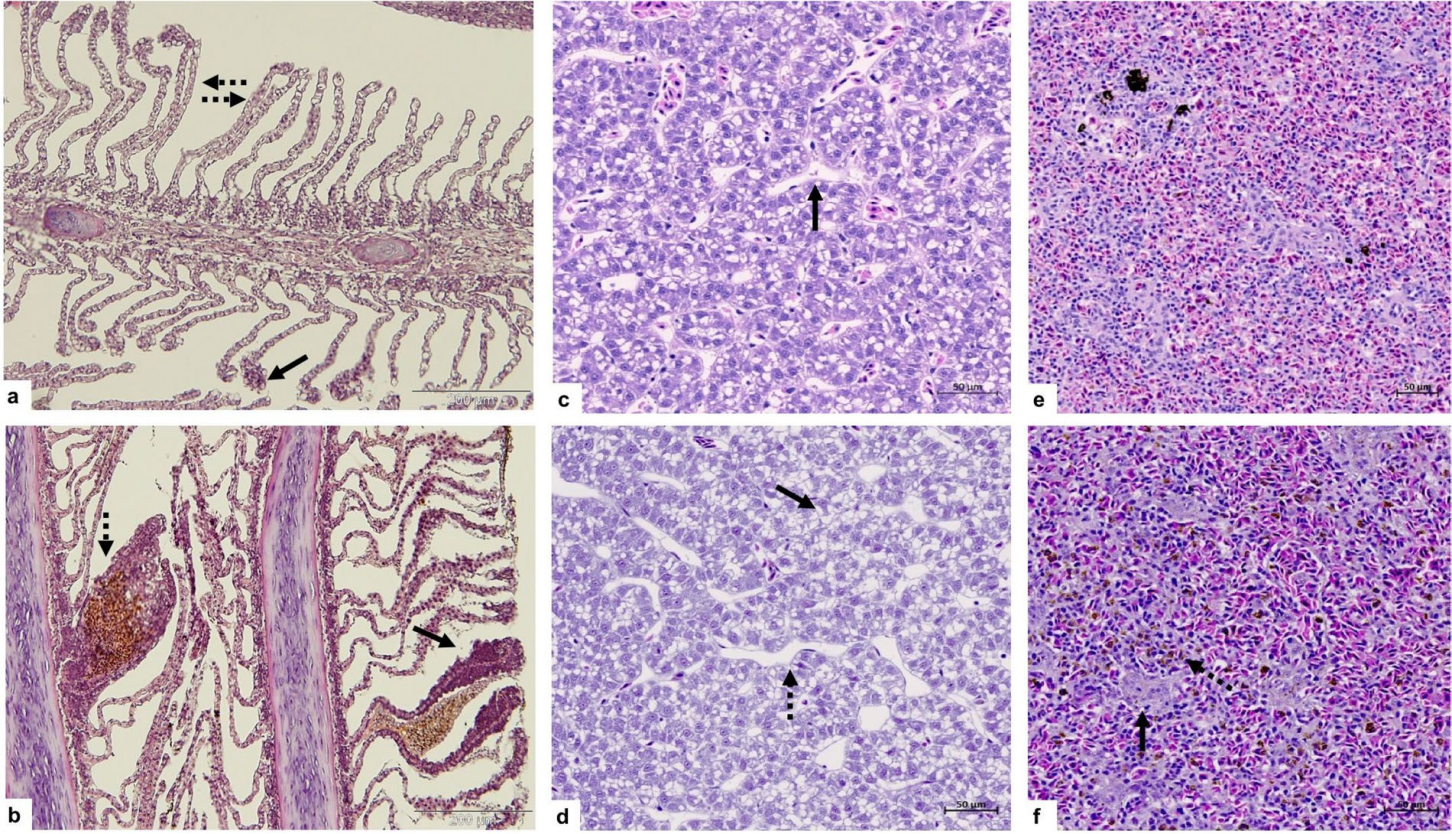
Pathohistological sections of gill tissue (**a,b**) from rainbow trout sampled before the treatment (**a**) and after three consecutive azamethiphos treatments (**b**). (**a**) Hyperplasia of lamellae pinnacles (arrow); Lamellar curling and lateral fusion (dotted arrows). (**b**) Severe dissociation of secondary lamellae mainly manifested as hypertrophy and hyperplasia of epithelial cells with curling (arrow); haemorrhage in between secondary lamellas, telangiectasia with clotted blood (dotted arrow). Liver tissue (**c,d**) from rainbow trout sampled before the treatment (**c**) and at the end of the third consecutive azamethiphos treatment (**d**). (**c**) Densely packed hepatocytes with dark stained cytoplasm; sinusoids filled with blood (arrow). (**d**) Areas with reduction in glycogen contents, focal necrosis and loss of hepatocyte integrity (arrow); distension and widening of an empty sinusoid (dotted arrow). Rainbow trout spleen (**e,f**) before the treatments (**e**) and after three consecutive azamethiphos treatments (**f**). (**e**) Normal histological structure of white and red pulp. (**f**) Granulomatous lesions (arrow) and presence of increased deposits of brown iron positive pigment (dotted arrow).

**Figure 6:**
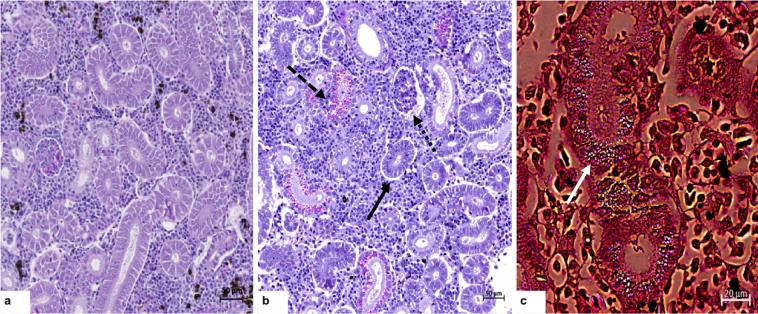
Kidney tissue from rainbow trout sampled before (**a**) and at the end of three consecutive anti sea lice treatments with azamethiphos bath (**b,c**). (**a**) Normal morphology with parietal epithelium of Bowman’s capsule, glomerulus, proximal and distal tubules and hematopoietic tissue. (**b**) Extensive peritubular dilatation (arrow), tubular degeneration, shrinkage of glomerulus and expansion of space inside Bowman’s capsule (dotted arrow), presence of hyaline droplets inside kidney proximal tubule epithelium cells (dashed arrow). (**c**) Histochemical detection of iron deposits (blue) within hyaline droplets and oxidative damage of tubular epithelium stained with Prussian blue.

The histological findings observed in the gill, liver, spleen and kidney of trout before (representing reference appearance) and after the series of azamethiphos treatments are shown in Figs 5 and 6. The gills from trout before the treatment showed normal anatomy of primary and secondary lamellae, with some mild alterations (+) (Fig. 5a). After the treatment, histopathological screening revealed moderate structural and functional alterations in gill tissue, extensively to the secondary lamellae with presence of severe haemorrhages (+++) (Fig. 5b). Liver from the fish before the start of azamethiphos treatments showed good condition with hepatocytes filled with glycogen (0) (Fig. 5c), whereas in the liver after the treatment, mild necrosis of hepatocytes (++) with severe dilatation of sinusoids (+++) and glycogen deposits were scarce (Fig. 5d). The spleen tissue from trout before the treatment showed normal appearance (0) (Fig. 5e), while after a series of delousing treatments excessive hemosiderin deposits (+++) and incidence of granulomatous lesions could be seen (++) (Fig. 5f). Kidney sections of the trout before the treatments showed a normal structure such as glomeruli and Bowman’s space with uniform renal tubules and interstitial hematopoietic tissue (0) (Fig. 6a). However, tissue damage of kidney after delousing treatments showed glomerular atrophy (++) and necrosis (++) (Fig. 6b), with an increased deposit of hemosiderin in proximal and distal tubules revealed using Pearls blue histochemistry method (+++) (Fig. 6c).

### Serum proteome analysis

Trout serum protein profiling yielded a total of 184 proteins. Potential indicators of azamethiphos induced impairment were identified from this list based on 3 criteria: (1) proteins that were present in each of the pooled sample; (2) representative proteins from each protein group (same name but different accession number); (3) proteins that were significantly altered between time point before, immediately after and 4 days after the azamethiphos treatment. A total of 47 (25.5%) proteins metal.l 3 criteria. This rigorous approach ensures that only the potential effects of the azamethiphos were identified and were present at higher or lower levels in azamethiphos exposed fish, with fold-changes ranging from 1.1 to 3.8. Protein identification following a MASCOT search is provided in Table 1. The major biological functions of altered proteins are involved in clot formation (e.g. fibrinogen, plasminogen, heparin, vitamin K-dependent protein), immune reaction (e.g. complement factors, histone), free heme and haemoglobin binding (e.g. haptoglobin, heme binding protein, beta globin) and lipid binding (e.g. apolipoproteins). Less representative processes affected are antimicrobial function (e.g. lg kappa chain, lysozyme) and body development (e.g. retinol, protein LEG1).

**Table 1:** List of proteins (with their main accession number, name and biological function) that were significantly altered in the serum of rainbow immediately after and 4 days after the treatment with azamethiphos, where (↓↑) indicates a fold change, compared to before the exposure time point.

Accession	Protein name	Coverage (%)	BEFORE/AFTER	BEFORE/4D AFTER	Main biological functions
NP_001117144.1	Beta-globin	78.23	2.0 ↑	1.3 ↑	Heme biding/oxygen transport
XP_014019752.1	Type-4 ice-structuring protein LS-12-like	65.52	1.1 ↓	1.3 ↓	Antifreeze protein
NP_001117138.1	Hemoglobin subunit beta	57.43	2.6 ↑	1.3 ↑	Iron ion binding/Oxygen transport
XP_014057057.1	Apolipoprotein A-IV-like	47.06	1.1 ↓	1.3 ↑	Lipid binding
XP_014004138.1	Uncharacterized protein LOC106573538	43.43	1.3 ↓	1.3 ↑	-
XP_014011079.1	Apolipoprotein C-I-like	42.53	1.6 ↑	1.3 ↑	Lipid binding
XP_014010562.1	Catechol O-methyltransferase domain-containing protein 1-like	41.70	1.1 ↓	1.3 ↑	O-methyltransferase activity
XP_014011035.1	Apolipoprotein A-I-like	41.18	1.1 ↓	1.3 ↑	Lipid binding
XP_014048452.1	Hemoglobin subunit alpha-4	37.06	3.2 ↑	1.3 ↑	Iron ion binding/Oxygen transport
NP_001117137.1	Serum albumin 1 precursor	31.25	1.5 ↓	1.3 ↑	Colloidal osmotic pressure of blood
XP_014000972.1	Lysozyme C II	29.86	1.0 -	1.3 ↑	Bacteriolytic
XP_014033195.1	Type-4 ice-structuring protein-like	27.50	1.1 ↑	1.3 ↑	Lipid binding
XP_014050741.1	Fibrinogen gamma chain-like	25.64	1.7 ↑	1.3 ↑	Protein binding/Inflammation/Clot formation
ACM09554.1	Ig kappa chain V region Mem5	19.58	1.1 ↑	1.3 ↑	Inflammatory response
ACN10174.1	Hemoglobin subunit alpha	19.57	2.3 ↑	1.3 ↑	Iron ion binding/Oxygen transport
XP_014060522.1	Protein LEG1 homolog	19.56	1.1 ↓	1.3 ↑	Organism developmet
ACM09183.1	Ig kappa chain V-IV region Len	16.18	1.2 ↑	1.2 ↓	Antimicrobial
XP_014053136.1	Heparin cofactor 2-like	13.84	1.0 -	1.3 ↑	Heparin binding
XP_013999410.1	Apolipoprotein B-100 isoform X2	13.46	1.1 ↑	1.3 ↑	Lipid binding
ACI66816.1	C-type lectin domain family 4 member E	13.25	1.4 ↓	1.3 ↓	Immune response/Calcium binding
XP_014039265.1	Complement factor I	13.13	1.4 ↓	1.3 ↑	Immune response
ACI66745.1	Plasma retinol-binding protein 1	11.98	1.2 ↓	1.2 ↑	Retinol transport
XP_014013823.1	Retinol-binding protein 4-B	11.98	1.1 ↓	1.3 ↑	Retinol transport
XP_014012275.1	Fibrinogen beta chain-like	11.73	2.1 ↑	1.3 ↑	Protein binding/Inflammation/Clot formation
XP_013986740.1	Complement C3-like	11.08	1.1 ↓	1.3 ↑	Immune response
ACI67052.1	Leukocyte cell-derived chemotaxin 2 precursor	10.26	1.1 ↓	1.1 ↑	Antimicrobial
XP_014042045.1	Alpha-2-macroglobulin-like	10.00	1.2 ↓	1.3 ↑	Serine protease inhibitor
NP_001133193.1	Beta-enolase	8.99	1.8 ↑	1.3 ↑	Magnesium ion binding
XP_014016495.1	Heparin cofactor 2	8.58	1.2 ↑	1.3 ↑	Heparin binding
XP_014019196.1	Haptoglobin-like	7.28	1.2 ↓	1.3 ↑	Free hemoglobin binding
XP_013997637.1	Apolipoprotein Eb-like	7.04	1.1 ↓	1.3 ↑	Lipid binding
XP_014069345.1	Vitamin K-dependent protein S-like isoform X1	6.54	1.1 ↓	1.3 ↑	Anticoagulation
XP_014061843.1	Fibrinogen alpha chain-like	6.22	2.6 ↑	1.3 ↑	Protein binding/Inflammatory/Clot formation
XP_014023990.1	Heme-binding protein 2-like	6.05	1.1 ↓	1.3 ↑	Heme biding
ACI69405.1	Histone H2B type 1-A	5.84	3.8 ↑	3.5 ↑	Inflammatory
XP_014003887.1	Complement C4-B	5.53	1.1 ↓	1.3 ↑	Immune response
XP_014048376.1	Complement c1q-like protein 2	5.46	1.5 ↑	1.3 ↑	Immune response
XP_014038830.1	Venom factor-like. partial	5.41	1.4 ↓	1.3 ↑	Lipid binding
XP_014060635.1	Plasminogen-like	3.48	1.2 ↑	1.3 ↑	Precursor of plasmin
XP_014055092.1	Saxitoxin and tetrodotoxin-binding protein 2-like	3.45	1.3 ↑	1.3 ↑	Toxin excretion
ACN10147.1	Ig kappa chain V-III region CLL precursor	3.20	1.4 ↑	1.3 ↑	Antimicrobial
XP_014070078.1	Alpha-2-antiplasmin-like isoform X1	3.17	1.4 ↓	1.3 ↑	Serine protease inhibitor
XP_013986847.1	Beta-2-glycoprotein 1-like	3.09	1.1 ↑	1.3 ↑	Phospholipid binding
XP_013979095.1	Histidine-rich glycoprotein-like	2.27	1.0 -	1.3 ↑	Cysteine-type endopeptidase inhibitor
XP_013996149.1	Complement factor B-like	1.86	1.7 ↓	1.3 ↑	Immune response
XP_013999413.1	Apolipoprotein B-100-like	1.39	1.1 ↓	1.3 ↑	Lipid binding
XP_014003484.1	Protein-methionine sulfoxide oxidase mical2b-like isoform X1	0.39	1.3 ↑	1.3 ↑	Flavin-adenine dinucleotide binding

Proteins are ranked in decreasing order of coverage (%).

**Figure 7:**
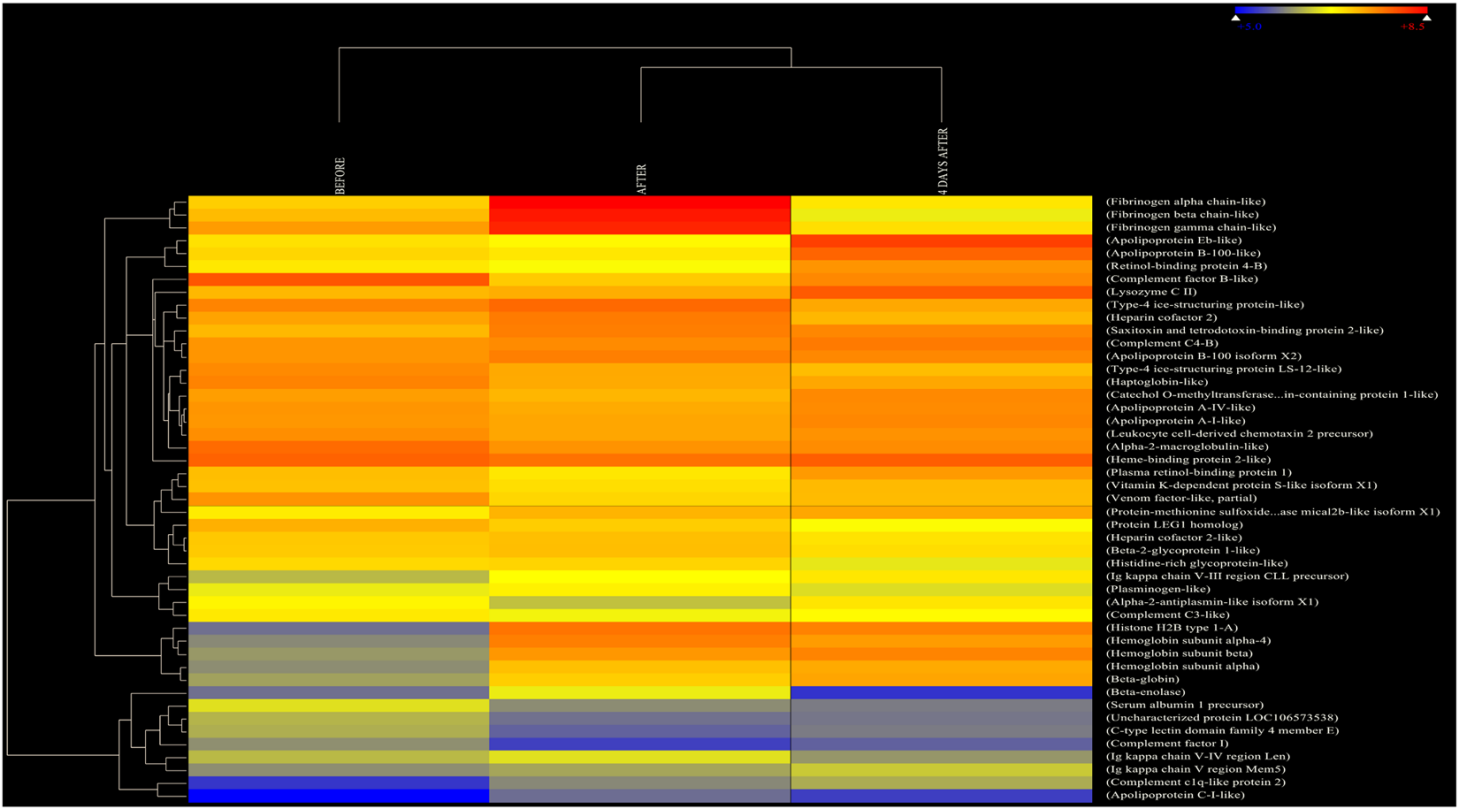
Effects of azamethiphos exposure on the serum proteome. Heat map showing hierarchical clustering of the serum proteomes for rainbow trout presenting the effects of the treatment immediately after and 4 days after the treatment on protein abundance.

Peak intensities at each time-point were analysed using Hierarchical Cluster Analysis to more clearly identify whether an association with treatment over time could be identified and to group proteins which possessed similar expression profiles (Fig. 7). The dendrogram at the top of Fig. 7 displays the connection of the protein abundances at the three time points and illustrates a clear distinction between those recorded before the treatment and those recorded immediately and 4 days after the treatment. Comparison of the proteome from before and immediately post treatment indicates a direct impact of azamethiphos. While comparison between before and 4 days post treatment indicates either a prolonged reaction pattern or homeostasis restoration. Table 1 shows the overall fold rise or decline in protein abundance for upregulated and downregulated (respectively) proteins before and after the treatment. Half of selected proteins (53%) were upregulated by the azamethiphos treatment in both sampling points post treatment. However, proteins such as complement factors, fibrinogen gamma chain, apolipoprotein, serum albumin, venom factor-like, antiplasmin, Ig kappa chains, protein-methionine sulfoxide oxidase, saxitoxin and tetrodotoxin-binding protein, macroglobulin, retinol-binding protein, catechol O-methyltransferase domain-containing protein, protein LEG1, leukocyte cell-derived chemotaxin precursor, heparin, plasminogen, haptoglobin like and heme-binding like protein showed a homeostasis restoration pattern. In contrast, only type-4 ice structuring protein and C-type lectin showed decreased abundancy in both time points after the exposure.

## Discussion

It is well established that treatments with anti-sea lice chemicals induces stress in rainbow trout^26–28^. Behavioural changes, as well as exhaustion and increased mortalities after repeated treatments were frequently reported by farmers. Sievers *et al*.^29^ reported 15% mortality in salmon 24 h after the treatment with 1 ppm AZA. Based on data collected from a commercial fish farm, we hypothesized that the cause of impaired fish health lies in the cumulative effect of repeated chemical anti-sea lice treatments. There is currently very little information on the toxicological impact on fish of the most commonly used anti-sea lice chemical (azamethiphos-AZA). To fill this knowledge gap a comprehensive investigation of the cumulative impacts of three consecutive treatments of azamethiphos in farmed rainbow trout was undertaken, to obtain an in-depth understanding of the effects on fish health through serum clinical chemistry changes with supportive histopathological alterations of fish tissues and serum proteome analysis.

Serum clinical chemistry has recently been described as a potential biomarker of chemical exposures in fish, particularly for organophosphates^30–32^. Serum proteome analysis is a relatively new method, and is now being frequently used in fish toxicological studies^33,34^. Moreover, negative effects of acute organophosphate exposure in fish can be detected at tissue level^35–37^, and novel findings in this area reveal the effects of organophosphates to haematology and red blood cell morphology^38–40^. This study is the first to investigate the effects of AZA in salmonids exposed to three treatments under farm conditions, where AZA was administered using short-term exposure by bath (45 min) at the recommended dose (0.2 ppm). Bioaccumulation of azamethiphos in salmon tissue is known to be very low and total azamethiphos is rapidly depleted after 10-degree days^41^. However, residue depletion was faster in muscle than in other tissues such as liver and skin^42^. After oral administration, azamethiphos is promptly excreted, primarily in the urine in form of 2-amino-3-hydroxy-5-chloropyridine as metabolite, which is then conjugated to glucuronic or sulfuric acid prior excretion. It has also been suggested that hepatic and serum carboxylesterases detoxify azamethipos^43^. No information has been found dealing with concentration of azamethiphos in fish blood after the treatment. In this study consecutive AZA treatments produced significant changes in the serum biochemical endpoints (Fig. 1). Higher concentrations of TP, ALB and CREA were observed in the early acute reaction immediately following the first and third treatments, as markers of increased liver function and impaired kidney tissue^30^.

In the late acute reaction (24 h and 48 h post treatment) a decrease in Cu concentration after the first treatment, and an increase in concentrations of CREA, Fe and HB during the second and third treatments were observed, along with a notable increase in TBIL concentration after the second treatment. Haemoglobin (HB) is the main protein in erythrocytes and it can be used as a marker of haemolysis, by measuring free HB in serum. The body’s primary source of Fe is from the turnover of haemoglobin in red blood cells. This is accomplished by spleen and liver macrophages which phagocytize RBC in pathophysiologic states such as haemolytic anaemia^44^. Once taken up by the macrophages, RBC are degraded in a phagolysosome which liberates haemoglobin, consisting of a heme group (porphyrin ring + iron) and globin chains. Once released from the heme group, iron must be converted to the Fe^2+^ state, which is facilitated by the copper-dependent enzymes^45^. This process is thought to be linked to the observed decreased Cu concentration during early acute reaction in first treatment and at 4 days after the second and third treatment. Bilirubin is the end product of heme metabolism and is released into the plasma, where it binds to albumin and is taken up by hepatocytes^46^. In the case of intravascular haemolysis, the released haemoglobin binds to haptoglobin and the complexes of haemoglobin-haptoglobin are ultimately cleared by spleen and liver macrophages^47^. For this reason, we measured the concentrations of haptoglobin and activity of glucose-6-phosphate dehydrogenase in serum at time points before, 48 h after and 4 days after the treatment. G-6-PD is an enzyme in the RBC hexose monophosphate pathway designed to produce reduced glutathione, that is used to avoid oxidative damage to haemoglobin and other intracellular structures^48^. In this study, we detected a significant increase in the antioxidant activity of G-6-PD, 4 days after the second treatment, which occurred after significant decrease of haptoglobin concentration 48 h after the same treatment, suggesting that haptoglobin was taken up and removed from circulation by free haemoglobin (Fig. 2b). This response is specific to the second treatment, occurring only 12 days after the first one.

The prolonged reaction period (4 and 10 days post treatment), showed a significantly higher concentration of TBIL and CREA in all three treatments and higher concentrations of Fe and P in first and second treatment. These biomarkers provide evidence of a moderately reduced glomerular filtration rate^30^ and further confirm the previously mentioned pathophysiological reaction following excessive red blood cell breakdown. Haptoglobin protective systems are saturated during severe serious haemolysis and glomerular filtration becomes the main pathway removing HB from circulation. Acute kidney injury, therefore remains as a significant complication after intravascular haemolysis^49^. Once released from the heme group iron can be stored within cells as ferritin, and over time becomes oxidized and degrades to form hemosiderin^50^. Hemosiderin can be visualized within macrophages as a dusky blue-grey pigment and can be definitively stained with Prussian blue (turning hemosiderin blue). In our study, fish kidneys showed tubule degeneration (cloudy swelling and hyaline droplets) which is the most common alteration found in the kidney of fishes exposed to water contaminants^51^. The presence of iron positive granules in proximate and distal tubules, coupled with the glomerular atrophy in this study indicates that the kidneys suffered from the damage caused by iron deposits at the end of the three consecutive AZA treatments (Fig. 6c). During these circumstances, renal damage mechanisms are regarded multifactorial and may result in direct damage of renal tubular cells through glomerular-filtered HB. Excessive hemosiderin deposits were also seen in spleen tissue after the treatments (Fig. 5f). Similar findings have been reported by Khan and Nag^52^ in plaice (*Pleuronectes platessa*) during haemolytic anaemia. These tissue changes observed in the liver correspond to previous findings from rainbow trout exposed to organophosphate chlorpyriphos^37^.

A possible explanation for the intravascular haemolysis caused by AZA arises from the fact that organophosphates inhibit erythrocyte cholinesterase activity and are also known to cause swelling, deformity and breakdown of the RBC^38,40^. Significant inhibition in erythrocyte acetylcholinesterase activity was observed in an 8-week lab based study in which groups of juvenile trout were exposed to sub-lethal concentrations (25 μg/L) of carbosulfan^53^. After one week of treatment, a 30% decrease in erythrocyte acetylcholinesterase activity was recorded. In a similar study with juvenile trout treated with biocide Chloramine-T, reduced activity of red blood cells AChE was evident following exposure to 30 mg of Chloramine-T after 3, 9 and 15 days^22^. Changes in the erythrocyte profile resulting from the acute impact of insecticide formothion in *Heteropneustes fossilis*^54^ and organophosphates such as quinalphos in *silver barb*^55^, malathion in *Cyprinus carpio*^56^ and trichlorphon in *Piaractus mesopotamicus*^57^ were reported. Still, evidence for AZA causing intravascular haemolysis in fish is scarce, despite a variety of studies reporting a toxic impact on marine invertebrates. Canty *et al*.^58^ suggest that AZA is relatively toxic to mussels following an exposure to 0.1 mg/L for periods up to 24 hours that resulted in a significant reduction in the activity of acetylcholinesterase in both haemolymph and gill, cell viability changes and decreased phagocytic index. Long-term (48 h) exposure of adult lobsters to azamethiphos concentration of 5 µg/L caused a significant change in haemolymph plasma biochemistry^59,60^.

The basic role of Principal Component Analysis (PCA) is to extract the main dimensions in the correlation structure of the analysed endpoints, with all endpoints having the same contribution to the overall variation. The first two dimensions encompass the main bulk of total variation, but does not necessarily include all of the most important endpoints. For this reason, despite CREA concentration showed a significant and systematic pattern on a univariate level, it is the least represented variable in the first two PCs. When looking at all three treatments together, CREA is not highly correlated with any of the other variables, so it is not significant when trying to explain the total variation in all endpoints (Fig. 3a). Interestingly, CREA is highly loaded on the third PC dimension, meaning that it is still important as a biomarker. In a similar way, variation in TBIL showed a pronounced shift in the second and third treatment, which is not well represented in the two PCA dimensions as these shifts do not correlate well with other variables. From the PCA analysis it was easy to distinguish well correlated pairs of biomarkers such as ALP and P, ALT and LDH, Cu and TP, Fe, HB and K. However, the mean data collected from the sampling point before each treatment (Fig. 3b) and for all sampling points for each treatment (Fig. 3c) show the cumulative effects on blood biochemistry, with weak overlap and complete segregation of the first treatment with other two treatments, respectively. This reduced separation of time points in later treatments (particularly following the second treatment) could be explained as a chronic effect following the more pronounced acute effect seen after the first treatment (Fig. 4a,c). Differences between biomarker average values for each treatment are illustrated by the mean lines in Fig. 1, with a significant decreasing trend seen in TP, ALB and K concentrations, and an increase in TBIL and P concentrations towards the end of the treatment period.

Serum protein analysis reveals an increase in beta-globin, haemoglobin subunits alpha, alpha-4 and beta, fibrinogen beta and alpha occurring immediately after treatment and persisting up to 4 days post treatment. Contrary to this, haptoglobin-like protein and heme-like binding protein were significantly decreased immediately after treatment and 4 days later were restored to pre-treatment levels (Fig. 7, Table 1). Proteomic analysis proved useful as a sensitive confirmatory method to support the previously mentioned hypothesis on excessive intravascular haemolysis in fish during repeated AZA treatments. Due to the expense involved in using this technique, proteome analysis was performed during the second (middle) treatment only. These techniques are not commonly used in aquaculture research, resulting in a lack of comparative results from this kind of analysis. The separation and identification of 47 proteins from trout serum that were altered following anti-sea lice treatment, will make a significant contribution to the use of these techniques in aquaculture research and the further understanding of the impact of sea lice treatment on salmonids.

## Conclusions

This is the first study to examine serum clinical chemistry in salmonids subjected to consecutive AZA treatments, enabling the investigation into activated biochemical and tissue pathways in trout. We also searched for alterations in the renal cortical tissue histology associated with haemoglobin (HB) excretion. Cumulatively, our results indicate that renal HB exposure has resulted in the accumulation of large amounts of oxidized ferric (Fe^3+^) in tissues and that these circumstances can foster heme-related cell damage pathway in tubule epithelial cells, characterized by an oxidative stress response. This study demonstrated that free heme is a probable cause for tubular barrier deregulation and oxidative cell damage and strengthened the hypothesis of uncontrolled free heme causing kidney injury, becoming observable with an increase in creatinine and phosphorus concentration after the treatments. These results also indicate that consecutive AZA treatments change the biochemical profile of trout, which could be explained through the physiological cascade of RBC destruction and elucidate potentially useful serum biomarkers for fish health monitoring during the anti-sea lice treatments. However, further studies are required to identify the particular mechanisms for AZA inhibitory action towards RBC acetylcholinesterase. In addition, the development of new and less aggressive substances to substitute the usage of organophosphates should become a priority for aquaculture research, together with usage of some nephron-protective agents as suggested by Georgiadis *et al*.^23^. We conclude that consecutive AZA treatments are causing cumulative impacts on fish health through a cascade of red blood cells intravascular degradation and accumulation of oxidized ferric (Fe^3+^) in different tissues of the rainbow trout. Therefore, we suggest, when possible, to use less invasive nonchemical anti-sea lice treatments and to secure sufficient intervals between repeated treatments to allow fish to recover from the previous treatment. Additionally, during recovery from the treatment it is important to keep the fish supplied with oxygen.

## Data Availability

The datasets generated during and/or analysed during the current study are available from the corresponding author on reasonable request.
