# Author Correction: Cumulative impact of anti-sea lice treatment (azamethiphos) on health status of Rainbow trout (*Oncorhynchus mykiss*, Walbaum 1792) in aquaculture

**DOI:** 10.1038/s41598-020-65512-0

**Published:** 2020-05-20

**Authors:** Josip Barisic, Stuart Cannon, Brian Quinn

**Affiliations:** 1 Aquaculture Health Laboratory, School of Health and Life Sciences, University of the West of Scotland, Paisley, PA1 2BE Scotland, UK; 2 Ruđer Bošković Institute, Division of Materials Chemistry, Laboratory for biotechnology in aquaculture, Zagreb, Croatia; 3 Kames Fish Farming Ltd., Kilmelford, PA34 4XA Scotland, UK

Correction to: *Scientific Reports* 10.1038/s41598-019-52636-1, published online 07 November 2019

This Article contains typographical errors. In the Materials and Methods section under subheading ‘Study location, sea lice treatments, sampling regime and water quality’,

“In summer 2017, after a sea lice infestation (>3 lice/fish), the pen under investigation was treated using Salmosan®Vet (azamethiphos, 500 mg/g powder) bath for three consecutive treatments, with 12 days between first and second treatment and 21 days between the second and third treatment. An enclosed plastic skirt (1800 m^3^) was used, and fish were treated with 0.2 ppm of azamethiphos for 45 min with additional oxygen injection as suggested by the manufacturer”.

should read:

“In summer 2017, after a sea lice infestation (>3 lice/fish), the pen under investigation was treated using chemical bath sea lice treatment containing the active ingredient azamethiphos for three consecutive treatments, with 12 days between first and second treatment and 21 days between the second and third treatment. A tarpaulin (1800 m^3^) was used, and fish were treated with 0.1 ppm of azamethiphos for 45 min with additional oxygen injection as suggested by the manufacturer”.

In the Results section under subheading ‘Seawater conditions and fish general health’,

“There were no immediate mortalities after the first two azamethiphos exposures, whereas mortalities occurred at the rate of 1% after the third exposure”.

should read:

“There were no immediate mortalities after the first two azamethiphos exposures, whereas mortalities occurred at the rate of 0.4% after the third exposure”.

